# The Interaction between Epigenetics, Nutrition and the Development of Cancer

**DOI:** 10.3390/nu7020922

**Published:** 2015-01-30

**Authors:** Karen S. Bishop, Lynnette R. Ferguson

**Affiliations:** 1 Auckland Cancer Society Research Centre, FM&HS, University of Auckland, Private Bag 92019, Auckland 1142, New Zealand; E-Mail: l.ferguson@auckland.ac.nz; 2 Discipline of Nutrition and Dietetics, University of Auckland, Private Bag 92019, Auckland 1142, New Zealand

**Keywords:** epigenetics, prostate cancer, breast cancer, colon cancer, methyl donors, nutrition

## Abstract

Unlike the genome, the epigenome can be modified and hence some epigenetic risk markers have the potential to be reversed. Such modifications take place by means of drugs, diet or environmental exposures. It is widely accepted that epigenetic modifications take place during early embryonic and primordial cell development, but it is also important that we gain an understanding of the potential for such changes later in life. These “later life” epigenetic modifications in response to dietary intervention are the focus of this paper. The epigenetic modifications investigated include DNA methylation, histone modifications and the influence of microRNAs. The epigenotype could be used not only to predict susceptibility to certain cancers but also to assess the effectiveness of dietary modifications to reduce such risk. The influence of diet or dietary components on epigenetic modifications and the impact on cancer initiation or progression has been assessed herein.

## 1. Introduction

The occurrence of cancer is dependent on the interplay between the genome and the epigenome, which together interact with environmental factors, including nutrition. The study of nutrition is complex as it is influenced by numerous variables that may have short (e.g., season) or long term (e.g., culture), subtle (e.g., exposure to marketing) or obvious effects (e.g., state of health or socioeconomic group). Despite these challenges, the influence of nutrition on epigenetics has been extensively studied [1,2,3,4], although many outstanding questions remain.

Epigenetics is the non-Mendelian inheritance of DNA modifications that may influence gene expression on one or more alleles, that is, epigenetic changes are heritable from cell to cell and may be heritable from parent to offspring [5]. Such epigenetic marks are acquired throughout life [6] and some are potentially reversible [7], but nonetheless, once established, are relatively stable [8].

It is widely accepted that there are critical windows during early development during which epigenetic marks are cleared and then re-established, and it is not surprising that an embryo would be particularly vulnerable to environmental influences during this time [9]. Although less pronounced, nutrition-induced epigenetic variation may occur throughout the life course [9]. Nutrition has trans-generational epigenetic effects, and more and more information is being gathered regarding when humans are most sensitive to nutritional epigenetic effects, and which nutritional components are likely to have the most profound impact. The Dutch Hunger Winter of 1944/5 and the Överkalix studies have been important in suggesting nutritional influences such as those of caloric intake, specific foods, nutrient and phytochemicals; as well as the importance of timing on the consequence of these epigenetic marks [1,2,3,10,11,12,13,14]. In pregnant woman the disease risk of their offspring varied depending on which trimester the foetus was exposed to the famine of the Dutch Hunger Winter [9]. Offspring exposed during the first trimester suffered more frequently from cardiovascular disease and reduced cognitive function later in life; those exposed during the second trimester tended to suffer with impaired kidney and lung function; whilst those exposed during the third trimester suffered more commonly from impaired glucose tolerance [9]. In addition, maternal exposure to the famine experienced during the Dutch Hunger Winter of 1944/5 immediately prior to conception, resulted in lower levels of DNA methylation in *insulin-like growth factor 2* (*IGF2)* (an imprinted gene coding for a growth factor expressed during early development) for fetuses compared to their same-sex siblings [11]. These epigenetic modifications persisted into old age and may have contributed to an increased incidence of obesity and late onset cardio vascular disease and/or diabetes [9,15]. However, although there is little to link this early life exposure to severe famine-induced *IGF2* hypomethylation to health status in adulthood, energy restriction during critical periods does appear to be associated with a reduced rate of colorectal cancer (CRC) [10]. Over the past two decades a number of case-control and prospective cohort studies were carried out to test the potential protective effect of various food-patterns, -groups and -components on the risk of developing a number of different cancers by modifying the epigenome [2,16,17,18]. Some of these effects and potential mechanisms will be discussed.

There is increasing attention on epigenetics, particularly as it is understood that genotype alone does not account for all cancer risk. It is widely accepted that many cancers could be avoided through changes in lifestyle. For example it has been suggested that approximately 45% of colon cancer cases could be avoided through diet and lifestyle changes [19]. In addition, it is useful to identify biomarkers for early signs of cancer development, since these can then be utilised to assess the potential benefit of a nutrient or food component for its effect on reducing cancer susceptibility [20]. Epigenetic modifications may qualify as such markers. An overview of epigenetics and the interplay between epigenetics, genetics and nutrition on the development of cancers, particularly breast, colon and colorectal cancers, are reviewed herein.

## 2. Epigenetic Modifications

Uncontrolled cell proliferation is one of the hallmarks of cancer, and loss of cell cycle control could be a contributory factor. Excessive caloric intake can increase uncontrolled cell proliferation, and dietary components that contribute to or inhibit excessive caloric intake and/or loss of cell cycle control may thereby play a role in the prevention of cancers.

In order to determine the impact of a particular food on epigenetic modifications and risk of a disease, the intake of the food of interest needs to be assessed retrospectively or prospectively in a suitable human population. To achieve this, a dietary intervention is often carried out. Before implementing a dietary intervention study it is first necessary to identify the dietary pattern, foods or food components to be included in the intervention [21]. Thereafter the intervention needs to be applied such that the study is sufficiently powered, and the data to be collected are identified. In prospective studies, communication with study participants occurs via printed material, face-to-face, telephonic, online or a combination of these methods. When implementing a dietary intervention it is imperative to assess adherence of the study participants to the proposed change in diet [22], as 100% compliance is an unrealistic expectation in a free-living environment. Compliance can be assessed using diet diaries, 24 h dietary recall, food frequency questionnaires and/or observation [22]. Urinary or blood biomarkers may also be used, and such biomarkers have the advantage of being objective. Each of these techniques has strengths and weaknesses regarding data collection and may include under reporting, recall bias, inconsistencies, observation bias, ease of administration and ease of data collection and analysis [23,24,25,26].

**Figure 1:**
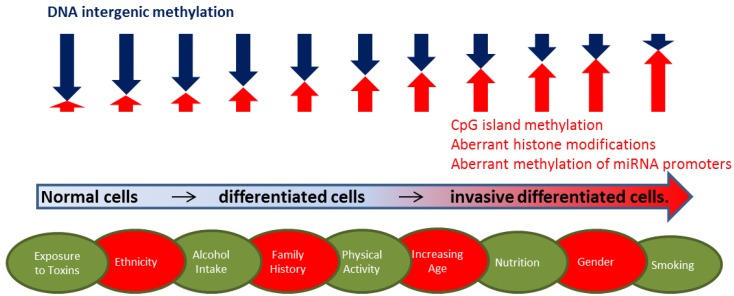
A diagrammatic representation of the extent and type of epigenetic modifications that promote cancer risk and/or progression, and the modifiable (in green ovals) and non-modifiable (in red ovals) factors that may influence these epigenetic modifications.

In addition to nutrition, epigenetic host factors contribute to the development of cancer. Such factors include DNA methylation, histone modification and the action of epigenetically modified small non-coding RNAs. DNA methylation is, to date, the most commonly reported epigenetic modification (which is not to say it is the most common); it is the most readily studied and this is likely the reason why more is known about it. DNA can be methylated sparsely throughout the genome at intergenic regions, or more densely at CpG islands that are often, but not exclusively located in promoter regions of tumour suppressor genes (TSGs), DNA repair genes or oncogenes [7]. The aberrant methylation of promoter regions can influence gene expression, and abnormal levels of global methylation are associated with numerous cancers (Figure 1). Histone tails can be post-transcriptionally modified, and these modifications (together with other epigenetic marks) determine whether the chromatin is active or inactive and this in turn affects the expression status of the genes within that chromatin region [7]. Small non-coding RNA can undergo DNA methylation or histone modification, thereby influencing the expression status of various genes. Before considering the interactions that may take place within the epigenome, genome and the environment with respect to the development of cancer, it is important to understand the types and roles of epigenetic marks we have detected to date.

Although there is evidence to support the role of dietary components in the regulation of epigenetically modified gene expression, the mechanism of action of these dietary components may vary amongst different cancer types [27]. An overview of the type of epigenetic modifications associated with cancers is provided below, followed by a discussion of the interactions between nutrition, the epigenome and cancer development and/or risk.

### 2.1. DNA Methylation

DNA methylation is a simple addition of a methyl group (CH_3_) to position 5 on the pyrimidine ring of the cytosine residue in a cytosine-guanine (CG) pair. Despite the potential mutagenic hazard of CpG dinucleotides, *i.e.*, they are involved in 35% of all point mutations leading to known genetic disorders in humans even though they make up only 1% of the human genome [28], CpG methylation is essential for life.

The genome of a young, healthy human is sparsely populated with CpG sites in intergenic regions and repetitive sequences, and many of these sites are methylated. In cancer, hypomethylation of these regions often takes place and hence the chromatin becomes less densely packaged and the DNA can be transcribed. Hypomethylation may occur in repetitive sequences or transposons, often leading to genome instability and DNA breakage. In addition, hypomethylation may bring about loss of imprinting control or demethylation of promoters that ordinarily would be silenced (e.g., the retrotransposon: *long interspersed nuclear element 1* gene), leading to cancers [29,30,31,32]. Loss of imprinting of *insulin like growth factor 2*, leading to microsatellite instability, was found to be associated with the development of CRC at a younger age [29], whilst Suter *et al.* suggested that hypomethylation of the L1 promoter was an early event in CRC [32].

In contrast to CpG sites in intergenic regions, promoter sites are frequently flanked by CpG dense regions, known as CpG islands and these islands are usually unmethylated. This lack of methylation helps ensure that the chromatin remains open and therefore the genes within that chromatin domain can be transcribed. In contrast, DNA hypermethylation of a gene promoter usually leads to gene silencing and this is one of the most common somatic aberrations in cancer [33]. During cancer therapy it is desirable to reactivate TSGs, and this can sometimes be achieved by demethylating TSG promoters [34]. Compounds such as 5-aza-2′-deoxycytidine (AZA), otherwise known as Decitabine, can be used for such a purpose [34]. AZA, and similar compounds, demethylate DNA by inhibiting DNA methyl transferases (DNMTs) [34,35], particularly DNMT3b [36]. DNMT3b is overexpressed in 30% of breast cancers and treatment with AZA has been found to return DNMT3b expression to normal levels in a breast cancer rat model [36]. Similarly, *RASSF1A* and *Trk* hypermethylation in hepatocellular carcinomas can be reversed following treatment with AZA, and normal gene expression restored [37,38]. Likewise, the promoter of the bromodomain protein, *BRD4* is hypermethylated in 31%–50% of colon cancers, and AZA can be used to demethylate the promoter resulting in re-expression of *BRD4* which impairs tumour growth [39]. However, in myeloid malignancies a complete response to AZA treatment is only seen in 10%–20% of patients despite reversal of TSG promoter hypermethylation [40]. Clearly inhibition of tumour growth is more complex than reversal of aberrant TSG promoter methylation.

A characteristic of human cancers is abnormal gene expression, and this change in expression can be brought about by both genetic and epigenetic changes in oncogenes and TSG [41]. In order to identify potential biomarkers Kim *et al.* assessed a number of candidate genes in a tumour panel and identified cancer-specific methylated genes associated with colon cancer [41]. *Oncostatin M receptor-*β (*OSMR*) was frequently hypermethylated in primary CRC tissues and matching DNA from stool samples. A decrease in *OSMR* expression was associated with progression of CRC such that more advanced disease, as indicated by tumour grade, was consistent with lower levels of gene expression [41]. Both Deng *et al.* and Ahmed *et al.* found that *OSMR* was hypermethylated in the majority of CRC tissues (90% and 78% respectively) [42,43] whilst Hibi *et al.* found only 32% of CRC tissues were hypermethylated. Unlike Kim *et al.* [41], Hibi *et al.* and Deng *et al.* found that hypermethylation was not associated with CRC progression [43,44]. Although dietary components have not been associated with a reduction in *OSMR* methylation, the authors speculate that this may be possible as both AZA and trichostatin A have been shown to increase *OSMR* gene expression [43].

In mammals, *de novo* DNA methylation takes place during embryogenesis and cell differentiation via the action of the DNMTs: DNMT3a and DNMT3b [45]. The established DNA methylation pattern is maintained during replication by DNMT1 [45]. If DNMT1 is absent, then passive demethylation takes place such that the amount of DNA methylation will halve each round of replication during the formation of daughter cells, leading to hypomethylation and aberrant gene expression. It is widely thought that dysregulation of the epigenome promotes the development of cancers and evidence continues to be published on the simultaneous progression in aberrant DNA methylation and advancement of cancers, particularly CRC [46]. The relevance of DNA methylation and DNMTs with respect to nutrition and cancer risk and/or progression will be discussed in Section 3.

### 2.2. Histone Modifications

Despite the importance of histone marks, much less is known about the influence of nutrition on histone modifications [20]. However, much is understood about the actual modifications. A nucleosome, which is the basic repeating unit in chromatin, allows DNA to be packaged within the nucleus. A nucleosome consists of double stranded DNA, 147 base pairs in length, wound twice around four histone proteins (H2A, H2B, H3 and H4) of two molecules each [47]. The nucleosomes are linked together by DNA of between 20–80 base pairs. Histone tails commonly extend from the *N* terminus of the histone proteins and the tails can be modified during embryonic development and throughout life [48]. Possible histone modifications include mono-, di-, and tri-methylation, acetylation, ubiquitylation, phosphorylation and ribosylation, and this most commonly occurs on the N terminus of histone tails [49]. Such modifications can influence the density of the chromatin, leading to a change in the accessibility of the DNA and hence its involvement in gene regulation [49]. For example, methylation on lysine 4 on the third tail of histone 3 (H3K4me3) usually leads to gene activation, whereas methylation of lysine 9 (H3K4me9) and/or 27 (H3K4me27) are associated with gene inactivation [50]. Only rarely is it one specific histone modification that determines gene expression levels [20], and therefore results can appear contradictory between studies depending on the histone modifications assessed. Instead, it is the combination of specific histones and specific types of modification. This makes the interpretation of histone modifications challenging. Commonly, histone methylation leads to a condensed chromatin structure and suppressed gene expression [51]. Just as lysine and arginine residues on histones can be methylated, lysine can also be acetylated. Histone acetylation usually leads to an open chromatin structure promoting gene expression [51]. Histone acetyl transferases are responsible for acetylation, and histone deactylases (HDACs) are responsible for the removal of acetyl groups. HDACs can target both histone and non-histone proteins such as transcription factors and DNA repair enzymes [52], so are important in the control of gene expression. Genes can be silenced by methylation of CpG islands and these genes can also be silenced by histone modifications without CpG methylation [53].

In breast cancers some histone lysine demethylases (KDM) are elevated (e.g., KDM5A) whilst others are expressed at low levels (e.g., KDM3B) [54]. Differential KDM expression is thought to be associated with aberrant histone methylation, particularly the demethylation of H3K4 [54]. Prognosis has also been associated with the expression levels of particular KDMs, for example low levels of KDM3B are correlated with shorter relapse-free survival [54]. In prostate cancer, several genes associated with DNA methylation and histone modifications have been found expressed at elevated levels [55]. In addition, increased H3K4diMe has been found in cancerous but not in normal prostate tissues and has been associated with risk of tumour recurrence [55,56]. H3K4diMe is correlated with activation of genes involved in cell proliferation and hence may influence tumourigenesis [55,56].

Some dietary components have a similar effect on HDACs as HDAC inhibitory drugs, and therefore might be useful in inducing cell cycle arrest or apoptosis in cancer cells [57,58]. These components will be discussed in Section 3.

### 2.3. Small Non-Coding RNA

Small non-coding RNAs, which make up much of the RNA content of a cell, are approximately 20–30 base pairs in length, and, as the name suggests, are RNAs that are not translated into protein. In spite of the latter, they are known to regulate gene expression in up to 30% of human genes and at least 60% of protein coding genes [50,59,60]. Small non-coding RNAs consist of microRNA (miRNA), piwi-interacting RNA (piRNA), small-interfering RNA (siRNA) and small nucleolar RNA. They act in regulating gene expression by a number of mechanisms, including heterochromatin formation and inhibition of translation [50,61]. In turn, epigenetic effects are known to regulate miRNA expression. Loss of acetylation of histones H3 and H4, as well as miRNAs that are transcribed from regions of DNA where the CpG islands are aberrantly methylated and therefore repressed, can cause silencing of the associated miRNAs [62]. For example, miRNA-124a is epigenetically silenced by hypermethylation in HCT116 (a colorectal cell line), and such hypermethylation has also been observed in cervical and gastric cancers [63,64]. This in turn leads to cyclin D kinase 6 overexpression, which is involved in cell-cycle progression [62]. Dysregulation of miRNAs is associated with the development of a number of cancers, and it is thought that miRNAs function as a genome surveillance mechanism [65].

There are a number of specific miRNAs that have been found to influence the expression of various genes. For example, partial methylation of the promoters of miRNA-29a and miRNA-1256 has been found in prostate cancer cells and tumours [66]. This partial methylation leads to decreased expression of these miRNAs [66]. This in turn results in the increased expression of *TRIM68* and *PGK-1* and these genes are associated with the progression of prostate cancer [66]. In breast cancer Qin *et al.* found statistically significant dysregulation of five miRNAs in malignant versus normal mammary tissue, namely miRNA’s -10a, -10b, -100, -145 and -205 [36]. MiRNA-10b was expressed at high levels in aggressive breast cancer, and was found to target the *homeobox D10* gene, which is a repressor gene involved in cell migration and invasion [67]. Aberrant promoter methylation or mutant *p53* can result in the down regulation of miRNA-145 expression due to the lack of p53-miRNA-145 binding in prostate cancer and numerous cancerous cell lines [68]. The atypical expression of numerous other miRNAs has been reported in a number of cancers, particularly metastatic cancers [62,69]. miRNAs show great potential for use as biomarkers and targets for therapy, particularly as aberrant epigenetic modifications can be reversed.

## 3. The Impact of Nutrition on Epigenetic Modifications and the Development and/or Progression of Cancers

In the following sections the influence of nutrition on different types of epigenetic modifications and the risk of progression of cancers are outlined. There are a number of dietary components that are considered influential in the development or inhibition of cancer (Table 1). These components include folate from green leafy vegetables, cinnamic acids from coffee, grain cereals, plums and kiwifruit, polyphenols such as epigallocatechin-3-gallate (EGCG) from green tea, resveratrol from red grapes and their products, sulforaphane and isothiocyanates from cruciferous vegetables, lignans from linseed, selenium and vitamin E. It is thought that many of these dietary compounds provide a protective effect against cancer by influencing epigenetic modifications [7]. Such nutritional effects may be organ specific [70].

**Table 1:** A summary of the epigenetic and anti-cancer effects of food components.

Food Component	Source	Epigenetic or Cellular Effect	Cancer Effect	Reference
Polyphenols: Genistein	Soybeans	Suppress expression of the androgen receptor (ER-β); inhibition of DMNT; demethylation of *RAR*β, *p16* and *MGMT* promoters; demethylation of promoters of miR-29a and miR-1256	Inhibition of PCa cell proliferation and invasion; decreased risk of PCa and breast cancer	[2,66,71,72,73,74]
Polyphenols: Resveratrol	Grapes, peanuts	DNMT 3b inhibitor; decrease in *RASSF-1*α methylation with increasing circulating resveratrol; Suppress expression of the androgen receptor	Decreased risk of PCa and breast cancer	[36,70,75]
Polyphenols: Epigallocatechin-3-gallate	Green tea	Demethylation and/or suppressed methylation of TSG promoters (*p15* and *p16*); inhibits HDAC activity.	Antioxidant activity; inhibition of angiogenesis; induction of apoptosis; inhibited invasive metastasis in a human pancreatic adenocarcinoma cell line.	[4,76,77,78,79]
Isothiocyanates	Cruciferous vegetables	Interaction with xenobiotic compounds, smoking and consumption of cruciferous vegetables	Anti-cancer effect: induced apoptosis and suppressed metastatic potential in lung cells.	[80,81,82]
Folate	Periconceptional folic acid supplementation; dark green leafy vegetables	Higher *IGF2* methylation in offspring; higher *hMLH1* promoter methylation	Lower birth weight; association with CRC risk.	[83,84]
Zinc	Seafood, beef, lamb	Zinc deficiency may induce protein kinase B and thus inhibit *PTEN* activity or inhibit alternative cancer associated inflammatory pathways.	Inhibition of cell proliferation in human prostatic carcinoma cell lines; evidence from cell line and mouse model studies (respectively): deficiency may contribute to prostate and oesophageal carcinomer risk and/or progression	[85,86,87]
α linoleic acid	Flaxseed	Decreased expression of COX 1 and COX 2 when fed to male Fischer rats; Decreased COX 2 expression when fed to hens; Changed expression of genes associated with brain	Tumour incidence, multiplicity and size decreased; reduction in ovarian cancer incidence and severity; influence on brain development.	[88,89,90,91]
		development, memory and learning in mice—no correlation between gene expression and methylation status; In mice, maternal supplementation induced hypomethylation of the *FADS2* promoter.		
Omega 3—EPA and DHA	Fish oils	Methylation of the COX 2 promoter in numerous cancer cell lines is linked to COX 2 silencing; Maternal intake of PUFA influences epigenetic regulation of FADS 2 in the offspring.	Fish oils increase apoptosis during tumour initiation and act through the COX 2 pathway; lower levels of COX 2 expression.	[92,93,94]
trans fatty acids	Industrially processed foods and low levels in meat.	DNA hypomethylation in the brains of offspring; histone modifications; hypomethylation at the SacII site in the *ER* gene in response to a diet high in omega 6 PUFA	during seven years of follow-up serum trans MUFA levels were associated with risk of invasive breast cancer.	[95]

*COX 2*: *cyclooxygenase-2*; CRC: colorectal cancer; DHA: docosahexaenoic acid; DMNT: DNA methyl transferase; EPA: eicosapentaenoic acid; *ER*: *Estrogen receptor*; FADS2: fatty acid desaturase 2; HDAC: histone deacetylase activity;* hMLH1:* *human mutL homolog 1*; *IGF2*:* insulin like growth factor 2*; *MGMT*: *O-6-methylguanine-DNA* methyltransferase; MUFA: monounsaturated fatty acids; PCa: prostate cancer; *PTEN*: *Phosphatase and tensin homolog*; PUFA: polyunsaturated fatty acids; *RARβ*:* Retinoic acid receptor beta*; *RASSF-1α*: *Ras association domain family 1 isoform α*; *TSG: tumour suppressor gene*.

The aforementioned three DNA modification mechanisms, namely methylation, histone modification and the action of small non-coding RNAs, interact to silence transposons and unpaired chromatin (e.g., sex chromosomes that are unable to pair with a perfect homolog), and thereby help maintain genome stability [65]. Aberrant epigenetic modifications and the risk of cancers both increase with age, with interactions of the genome and epigenome as well as with environmental factors such as diet type likely contributing to cancer risk [2,6].

The most sensitive time in the epigenome is during primordial germ cell development and early embryo development, *i.e.*, times during which epigenetic marks are cleared and re-established. From the Dutch Hunger Winter 1944/45 and the Överkalix studies, amongst others [96], we know that nutrition and lifestyle habits during gestation and early adolescence can have an impact on the health of the adult by modifying their epigenetic profile. However, there is also evidence that nutrition later in life can influence health with respect to the development of cancers. Such evidence is largely presented through performing dietary intervention studies and assessing the effect of food components on epigenetic modifications in both human [97] and animal models [79]; evaluating the association between TSG methylation status and presence of disease [36,64,98]; and either the level of blood markers of dietary intake and/or self-reported intake and association with disease risk [99,100].

Cancer is caused by an imbalance in the mechanisms that control cell proliferation. The loss of control of cell proliferation can be due to genetic mutations and epigenetic aberrations, many of which accumulate over time. There are limitations associated with both epidemiological and intervention studies. Epidemiological studies, for example, cannot be used to differentiate between cause and effect; differences in genotype and lifestyle may cloud relatively small size effects due to diet; and cancer prevention trials take time depending on duration of cancer development [20]. Nonetheless, there are studies that convincingly show both early life and later life nutritional effects on the epigenome.

### 3.1. The Impact of Folate on Epigenetic Modifications Associated with Cancer

Folate is an important one-carbon donor, and one-carbon metabolism is essential for the synthesis of DNA, proteins and phospholipids [101]. Folate is obtained solely from the diet and is converted to 5,10-methylenetetrahydrofolate (MTHF). MTHF acts as a methyl donor, converting homocysteine to methionine and methylating DNA [20]. Methyl groups from methionine and choline are used to form S-adenosyl-methionine (SAM), which is an important DNA methylating agent [102]. Deficiency in folate does not have the same impact in all tissues and at all stages of development. For example; Chang *et al.* showed an increased risk of offspring with neural tube defects born to mothers with low serum folate levels [103]. Similarly, those with the *MTHFD1* G1958A genotype were found to be at higher risk for giving birth to babies with a neural tube defect, thought to be due to a greater demand for choline (for example, from eggs) as a methyl donor [102].

Folate deficiency is thought to exert its effect through a number of possible mechanisms namely: uracil misincorporation (*i.e.*, inducing mutations), inhibiting DNMT1, and promoting aberrant global and promoter methylation [7,104,105,106]. However, much of this work has been carried out in mouse models and therefore may not apply directly to humans [107,108]. The associations between folate and epigenetic modifications appear somewhat inconsistent, since they are dependent on the cell type, epigenotype and genotype [109,110,111] and may vary depending on whether supplemental or dietary folate was consumed [107]. It is apparent that adding extra folate to the diet may exert different effects depending on the amount consumed, the stage of development during which it is consumed [104,112] and the genetic and epigenetic background of the person taking it. For example, people with the *MTHF reductase* 677TT genotype and a high intake of alcohol are at greater risk of aberrant methylation of cancer-related genes (e.g., *RASSF-1*α) and an increased risk of oral squamous cell carcinoma [113] which could be exacerbated by low folate levels [114]. It is widely accepted that elevated ethanol consumption interferes with the production of SAM by inhibiting the availability of vitamin B6 and B12 [9], and therefore it is not surprising that risk of oral squamous cell carcinoma is acerbated by high alcohol and low folate intake.

In the Netherlands Cohort study the methylation status of the promoters at specific genes involved in the development of colorectal cancer (*APC*,* p14*,* p16*, *hMLH1 and RASSF-1*α) was investigated in adults [115]. Similar to the findings in oral squamous cell carcinoma [114], an association was found between an increased level of methylation in these gene promoters and people with low folate and high alcohol intake (relative to a high folate and low alcohol intake) [115]. This finding is also consistent with data published by Supic *et al.* [113]. Whilst folate supplementation can be beneficial, it can also be harmful, as high serum folate levels may be detrimental in those who already harbour neoplastic lesions [84]. This is consistent with the role of folate derivatives as cofactors in nucleotide synthesis. Folate also has the potential to promote rapid cell proliferation [84]. Although it has been shown that folate deficiency can induce decreased DNA methylation in older women, a delayed response was seen in folate repletion [97]. In a Chinese study, a higher intake of folate was associated with a decreased breast cancer risk in pre- rather than post-menopausal women [116], suggesting that timing of a dietary intervention may elicit different effects. It is clear that folate deficiency can mediate carcinogenesis [7] but conversely increasing serum levels of folate may lower the risk of CRC [117] or increase the risk of aberrant methylation in people with previous CRC [104].

### 3.2. The Impact of Polyphenols on Epigenetic Modifications Associated with Cancer

Polyphenols act as antioxidants, and are found abundantly in foods originating from plants. Polyphenols consist predominantly of four main types, namely phenolic acids, benzoic acids, stilbenes and flavonoids [98,118]. Polyphenols are found in foodstuffs such as green tea, coffee, red wine, soy, vegetables (e.g., onions, asparagus and carrots) and fruits (e.g., grapes, citrus fruits, berries and apples), amongst others. However, the most abundant polyphenols are not necessarily those with the highest bioavailability [118].

#### 3.2.1. Green Tea

Green tea polyphenols have been shown to inhibit tumour invasion and angiogenesis in a skin cancer mouse model [119]. The regular consumption of EGCG, a green tea polyphenol, or green tea itself may also decrease the risk of CRC, oesophageal, breast, hepatocellular, ovarian, pancreatic and prostate cancer development in adults [79,99,100,120]. EGCG has been found to have both epigenetic effects in humans and in carcinoma cell line studies through demethylation or suppressed methylation of TSG promoters [4,77,78]. It can also exhibit anti-cancer activity via anti-oxidant effects [76] depending on the type of cancer.

The consumption of foods high in EGCG may also have an impact on histone modifications. In a pancreatic cancer mouse model, Kim and Kim [79] showed that EGCG inhibited invasive metastasis via the inhibition of HDAC activity (through the regulation of Raf kinase inhibitor protein) and increased histone H3 expression.

#### 3.2.2. Resveratrol

The epigenetic effects of resveratrol have been assessed in a number of cancer models. Qin *et al*. evaluated the effect of resveratrol at two different concentrations on breast cancer in a rat model and found that expression of DNMT3b decreased in the tumour cells but increased in the cells from normal tissue [36]. In addition, treatment with high levels of resveratrol dysregulated a number of miRNAs (miRNA21, -129, -204 and -489) in tumour but not in normal tissues [36]. In women at high risk of developing breast cancer, circulating levels of resveratrol, rather than actual dosage received, was found to reduce the methylation of *Ras association domain family 1 isoform* α (*RASSF-1*α), a TSG, but did not change the methylation status of other genes assessed [75]. However, resveratrol did not affect the methylation pattern of *RASSF-1*α in MCF7 breast cancer cells [121].

Resveratrol may modify histone acetylation activity as it is a *Silent Information Regulator 1* (*SIRT1*) activator [122]*.* SIRT1 is important as it has HDAC activity and thus can regulate the transcriptional activation or repression of a number of genes, including *p53* [122] and thus resveratrol may protect against cancer. Resveratrol is non-toxic at high doses and is rapidly metabolised, yet concentrations may be low in serum and higher in other tissues such as the colon [123]. Due to the anti-cancer activity of resveratrol, efforts are currently focused on modifying the resveratrol backbone to increase bioavailability and biological activity [123]. The impact of such changes to the backbone of resveratrol on histone modifications remains to be seen.

#### 3.2.3. Caffeic Acid

Caffeic acid, another dietary polyphenol, affects the bioavailability of SAM. As mentioned previously, SAM acts as a universal methyl donor and is therefore required for methylation. Dietary polyphenols usually inhibit the maintenance of methylation by interacting with the catalytic site of DNMT1 [124]. In a population-based study Geybels *et al.* found that a reduced risk of recurrent or progressive prostate cancer was associated with the consumption of coffee, but not with tea [125]. In a review by De *et al.* [126] it was concluded that caffeic acid suppresses ultraviolet B-induced *cyclooxygenase 2 (COX 2)* expression by binding to *Fyn* and blocking Fyn kinase activity. *Fyn* is an oncogene of the protein tyrosine kinase family, and blocking Fyn kinase activity is believed to reduce the risk of skin cancers [126].

#### 3.2.4. Genistein/Daidzein

Soy contains the isoflavones genistein and daidzein [127]. Isoflavones may influence cancer risk as they behave like estrogens and thus act on the estrogen receptor. Genistein binds to the active site of the estrogen receptor, and has been found to inhibit the growth of estrogen negative breast cancer [128]. However, genistein may be contra-indicated in women with estrogen positive breast cancer, particularly those receiving Tamoxifen [128]. The intake of soya during childhood [129] as well as consumption during adolescence [130] has been associated with a decreased risk of breast cancer. In a study of premenopausal women supplemented with isoflavones (a combination of genistein, daidzein and glycitein), the change in methylation of five cancer related genes was assessed [131]. This isoflavone mix induced increased dose related methylation in the proliferation regulatory genes associated with breast cancer development, *retinoic acid receptor* β*2* (*RAR*β*2*) and *cyclin D2* (*CCND2*) [131]. In prostate cancer the partial methylation of the aforementioned promoters down-regulates the expression of *RAR*β*2* and *CCND2*, thereby increasing the expression of *tripartite motif containing 68* (*TRIM68)* and *phosphoglycerate kinase 1 (PGK1)*, which in turn promotes cell proliferation and invasion [66]. Genistein inhibits DNMT1 and hence, not surprisingly, Fang *et al.* found a reversal of the hypermethylation found in *RARB*,* p16* and *MGMT* gene promoters in prostate, breast and oesophageal cancer cell lines [71] thereby reactivating these epigenetically silenced genes.

Genistein has been found to demethylate partially methylated promoters of miRNA-29a and miRNA-1256 in prostate cancer cell lines [66]. Demethylation of these promoters leads to the decrease in expression of *TRIM68* and* PGK1* in prostate cancer cells, whilst methylation of the promoter of miRNA-29a led to increased expression of *Myeloid cell leukemia sequence 1* (*Mcl-1*) which blocks apoptosis and supports cell survival in lymphoma [132]. In a mouse model increased expression of miRNA-29a led to decreased expression of *Mcl-1*, which resulted in reduced tumour growth [132].* TRIM68* and *PGK1* promote the inhibition of prostate cancer cell proliferation and invasion [66], thus reducing the risk of metastasis.

#### 3.2.5. Selenium

In addition to polyphenols, other food components may also play a role in establishing or modifying the histone epigenome. One such component is selenium. Selenium supplementation has been linked to an apparent decrease in cancers in general whilst other associations such as the protective effect against lung and breast cancers, are more controversial [133,134]. Epidemiological studies and clinical trials have shown a benefit with respect to a decreased risk of developing prostate cancer when diets were supplemented with selenium [134]. For this particular study selenium enriched yeast was used, and this contains selenomethionine, the form of selenium that is naturally found in foods such as grains, onions and broccoli [134]. However, the selenium content of plants is largely dependent on the selenium content of the soil on which it is grown.

Selenium enriched yeast has also been shown to inhibit the proliferation of prostate cancer cells and breast cancer cells in mouse models [135,136]. There has been controversy regarding the potential anticancer benefits of supplementary selenium. The apparent variability of outcomes of human selenium supplementation trials may be partly due to the variability of baseline selenium levels of the cohort under study, since too much selenium can be as hazardous as too little. The form of selenium used in the intervention also appears to be important [133,137]. Butyrate induces DNA methylation and acts as an inhibitor of histone deactylase, and α-keto-γ-methylselenobutyrate (a deaminated product of selenium-enriched yeast) resembles butyrate. Therefore selenium enriched yeast and selenomethionine could act as prodrug inhibitors of histone deactylases [7,138], which may assist in the prevention of aberrant histone methylation.

#### 3.2.6. Isothiocyanates

Isothiocyanate (ITC) is a type of degraded glucosinolate that is bioactive and believed to induce anti-carcinogenic effects [80]. Glucosinolates and sulforaphane are dietary isothiocyanates found in cruciferous vegetables such as broccoli and brussels sprouts. An association has been found between cruciferous vegetable intake and a reduced risk of various cancers (or level of biomarkers for specific cancers) in a number of human, animal and *in vitro* studies detailed as follows [17,139,140,141]: treatment of colon cancer cells with sulforaphane suppressed the expression of DNMT1 and increased the expression of genes involved in regulating cell cycle arrest and decreasing cell proliferation [141], and thus lowering cancer susceptibility. Sulforaphane was observed to have an inhibitory effect on histone acetylation by inducing depletion of HDAC3 in human colon cancer cells [57]. This effect was found to be reversible following removal of sulforaphane from the culture medium [57].

Using prostate cancer cell lines Hsu *et al.* [142] showed that sulforaphane down regulated the expression of both DNMT1 and -3b, leading to demethylation of the *CCND2* promoter and restoration of gene expression. CCND2 is a regulator of cell cycle and can thereby exert anti-cancer effects [142]. In an *in vitro* study, sulforophane and green tea polyphenols were used to reactivate the estrogen receptor in an estrogen receptor negative breast cancer cell line [143], and thereby increasing sensitivity to Tamoxifen, which is an estrogen receptor modulator. When treated with sulforaphane, green tea polyphenols and Tamoxifen, an increase in cell death and an inhibition of cell proliferation was noted when compared with the effect of Tamoxifen alone [143]. In addition, with combined treatment with sulforaphane and green tea polyphenols, hypomethylation of the ER promoter took place, as did inhibition of DNMT activity and enhanced HDAC inhibitory activity [143].

#### 3.2.7. Vitamin D

In addition to selenium, vitamin D may also play a role in reversing aberrant epigenetic modifications. There is a known interaction between vitamin D and the epigenome, but the interaction with breast cancer remains unclear [20]. Although there is some evidence to suggest there is an interaction between vitamin D levels and modification of the epigenome that might have an impact on the development of breast cancer [144], further work needs to be carried out to expand on these findings. The active form of vitamin D, namely calcitriol, promotes cell cycle arrest and induces apoptosis (through the upregulation of MKK1, an apoptotic signaling molecule) thus exerting an anti-tumour effect [145,146]. Levels of calcitriol can be maximized by the administration of pharmacological doses and/or by inhibiting the expression of CYP24, which catabolizes vitamin D analogues [146]. 1α-hydroxylase expression, which is important for the production of the active form of vitamin D, is modified by HDACs. In addition, the *vitamin D receptor* (*VDR*) gene can impact on this HDAC activity of 1α-hydroxylase if the *VDR* promoter is DNA methylated [147]. Aberrant methylation of gene promoters has frequently been implicated in the development and/or progression of various cancers, for example the *tropomyosin-related kinase (Trk)* gene promoters in medullary thyroid carcinoma [148], hepatocellular carcinoma [37] and ovarian cancer [149]; and prior to the development of breast cancer, the *RAR*β*2* gene is often silenced through demethylation of the promoter region [150]. Consistent with this theme, there is evidence to support the notion that the methylation of the *vitamin D receptor* gene promoter may contribute to the development of prostate cancer as decreased expression of 24-hydroxylase mediates the first step in the degradation of 1,25(OH)_2_D_3_, which is associated with prostate cancer risk [145,151]. However, epigenetic modifications influencing the impact of vitamin D on cancer risk and development are complex and largely unexplored.

#### 3.2.8. Lycopene

Lycopene, a type of terpenoid, is also thought to influence the risk and/or progression of cancers but evidence of the mechanisms of action is not sufficient to draw conclusions with respect to ability to modify methylation status. However, lycopene appears to partially demethylate the TSG *glutathione S transferase P1* in breast cancer cells and *RAR*β*2* and* harpin induced 1* (possible early markers for cancer) genes in non-cancer breast cells and therefore may regulate gene expression [152].

## 4. Conclusions

Certain diets are widely accepted as being associated with risk for particular cancers, for example, increased risk of colon cancer is associated with diets high in animal fats, whilst lower risk is associated with a diet high in fresh fruit and vegetables, legumes and oily fish [153]. These associations provide us with a starting point with respect to which foods might modify the epigenome and the genes that are likely to be influenced. Cancers, such as colon and prostate cancer can take decades to develop and the authors agree with the proposal from Burdge *et al.* [154] that perhaps it is time we considered a “life course” perspective on cancer development. If we were to focus on the diagnosis of a high risk epigenetic state, *i.e.*, by defining a protective epigenetic profile for cancers in general or a specific cancer, it may be possible to assess the impact of interventions and more clearly understand the interactions between nutrition, the epigenome and cancer development. It would also be important to increase the focus on targeting diet during susceptible epigenetic periods. Teegarden *et al*. discussed the importance of identifying *in utero* exposure and gene expression profiles together with nutrition and epigenetic profiles associated with breast cancer [20]. An understanding of epigenetic mechanisms in the development and progression of cancers will not only allow us to identify various high risk profiles and enable us to monitor response to treatment, but may also provide a platform from which we can design lifestyle interventions. The identification of high risk profiles will likely involve multicenter longitudinal studies to enable the epigenome to be assessed at a number of time points, together with the collection of data on dietary intake and development of disease. Ideally advantage would be taken of studies that commenced at birth with the possibility of continuation, such as the Northern Finland Birth Cohort study [155] and the Growing Up in New Zealand study [156]. Incidence and mortality from cancer is escalating in low and middle income countries, and cancers are likely to be the next biggest challenge in these countries [153]. With this in mind, it is imperative to understand the implications of diet on epigenetic modifications, and the effect of those modifications on the development of cancer today and in future generations. Such an understanding and an appropriate resultant response would help decrease the level of risk in future generations.
